# Semantic-Structure-Aware Multi-Level Information Fusion for Robust Global Orientation Optimization of Autonomous Mobile Robots

**DOI:** 10.3390/s23031125

**Published:** 2023-01-18

**Authors:** Guofei Xiang, Songyi Dian, Ning Zhao, Guodong Wang

**Affiliations:** 1 College of Electrical Engineering, Sichuan University, Chengdu 610065, China; 2 National Key Laboratory of Special Vehicle Design and Manufacturing Integration Technology, Baotou 014031, China

**Keywords:** simultaneous localization and mapping (SLAM), semantic, information fusion, orientation estimation, mobile robots

## Abstract

Multi-camera-based simultaneous localization and mapping (SLAM) has been widely applied in various mobile robots under uncertain or unknown environments to accomplish tasks autonomously. However, the conventional purely data-driven feature extraction methods cannot utilize the rich semantic information in the environment, which leads to the performance of the SLAM system being susceptible to various interferences. In this work, we present a semantic-aware multi-level information fusion scheme for robust global orientation estimation. Specifically, a visual semantic perception system based on the synthesized surround view image is proposed for the multi-eye surround vision system widely used in mobile robots, which is used to obtain the visual semantic information required for SLAM tasks. The original multi-eye image was first transformed to the synthesized surround view image, and the passable space was extracted with the help of the semantic segmentation network model as a mask for feature extraction; moreover, the hybrid edge information was extracted to effectively eliminate the distorted edges by further using the distortion characteristics of the reverse perspective projection process. Then, the hybrid semantic information was used for robust global orientation estimation; thus, better localization performance was obtained. The experiments on an intelligent vehicle, which was used for automated valet parking both in indoor and outdoor scenes, showed that the proposed hybrid multi-level information fusion method achieved at least a 10-percent improvement in comparison with other edge segmentation methods, the average orientation estimation error being between 1 and 2 degrees, much smaller than other methods, and the trajectory drift value of the proposed method was much smaller than that of other methods.

## 1. Introduction

With the rapid development of sensing, computation, manufacturing, and control technologies in recent years, various kinds of robots have been coming into our lives and work gradually, such as unmanned aerial vehicles, robot vacuum cleaners, intelligent vehicles, autonomous disinfection robots, logistics delivery robots, and so on. These different kinds of robots have been transforming our social lives ever-increasingly [1,2]. For example, during the current COVID-19 pandemic, we always expect to use robots to replace humans to complete the disinfection work in public places, due to different regions in the environment having different risks. Thus, in these environments, one of the most-important prerequisites for the robots to accomplish the task safely, autonomously, and efficiently is that the robot should know its own location relative to the environment. Therefore, it is of great importance to endow the robot with the ability of autonomous navigation. Simultaneous localization and mapping (SLAM) [3] is such a technique that can build the environment map and compute the location in the map simultaneously. With the help of SLAM, when a robot enters an uncertain or even unknown environment, it can make use of the structured environment information to determine its location on the map; it can also reconstruct its surroundings by only relying on its own sensors. Thus, the robot can move and complete specific tasks in a prior unknown and unstructured environment autonomously [4,5].

SLAM has been widely applied in various applications. Since the robot has to build the environment map and locate its positions by the sensors carried by it, the most-fundamental problem is how to accurately and robustly extract information that can be used for map building and localization. Conventionally, the information extraction techniques have been utilized to obtainfeatures directly from the available sensors by some manual rule-based methods, then these features will be used for map building and localization. With the rapid development of deep-neural-network-based learning methods, we can obtain much high-level semantic information from the environment by the nature of the multi-level information processing mechanism. Thus, the accuracy and robustness of the SLAM system can be improved further by this high-level semantic information [3,6,7].

Semantic information plays a positive role in the SLAM system of mobile robots. The semantic information can enhance the robustness against those disturbances in the environment and systems [8,9,10]. During the computing of semantic information, the effective information generally flows from low-level, low-accuracy to high-level, high-accuracy by the nature of the hierarchical structure of the deep neural network; thus, this high-level information can adapt to various variations in the environment [11,12,13,14]. Some works showed that the stability of the features can be enhanced by filtering out those features of dynamic or potentially moving objects. Some works showed that this high-level semantic information lowers the sensitivity to sensor noise to some extent, such as straight lines, triangles, and planes, which are more robust than the original sensor data [15,16]. Some work showed that semantic information can improve the accuracy and reliability of data association by taking advantage of relevance, heterogeneity, and invariance in semantics [17,18]. On the other hand, semantic information can support the accomplishment of various high-level tasks to a great extent, since these high-level tasks are usually encoded by some natural-language-like semantics, consisting of some specific, abstract symbols, logic, and temporal operators, such as linear temporal logic, signal temporal logic, metric temporal logic, and so on [19,20,21,22]. However, though semantic SLAM has achieved state-of-the-art performance in various practical applications, the purely data-driven-based neural network model has also shown many disadvantages, such as the non-explainability issue and the huge amount of labeled data requirement, both of which limit its adaptability in different environments, especially the human beings involved and safety-critical scenarios [23,24]. Therefore, in this work, we hope to resolve the aforementioned problem partially by embedding rule-based and explainable structures, allowing the robot to be able to adapt to the new environment efficiently.

In practical applications, in order to be able to achieve the all-around perceptual coverage of objects in any direction in the task scene, mobile robots often need to be equipped with multiple vision sensors to meet this demand. In smart cars, for example, the vision system on board often consists of multiple high-resolution cameras with different viewing angles to cover a 360-degree field of view around the vehicle. Having a wider field of view means that the robots can observe more information about the environment, but it also means that the amount of computation required to process this information is greatly increased. In addition, under the current situation that the perception method based on the deep neural network model has become the mainstream, more raw perception data input also require corresponding orders of magnitude labeled data, which undoubtedly greatly increases the cost and time of the perception model when adapting to new scenarios, which is not conducive to rapid deployment and application in new scenarios.

In summary, the main contributions of this article are summarized as follows:

- We propose a multi-level semantic-structure-aware global orientation estimation framework, which consists of a semantic information extraction module and a global orientation estimation module.
- In the semantic information extraction stage, we attempted to process the surround view synthesized images obtained after the inverse perspective mapping (IPM) of the original image and use the passable area segmentation mode, which more easily obtains the annotation data, fully combines the potential prior information in the task, and obtains the boundary including the passable area. The semantic information including visual feature points and ground marking edges in the passable area can effectively reduce the requirements of the semantic perception model for labeled data and, finally, meet the needs for tasks such as mapping and positioning.
- In the orientation estimation stage, we designed a segmentation method for marker lines based on the structural rules of the Manhattan world, which can be used to obtain from the image a collection of line segments that conform to the structural assumptions of Manhattan and the dominant orientation of these lines; thus, we can distinguish the marker lines from the noise lines.
- We validated the effectiveness of the proposed scheme by taking the semantic perception task of intelligent vehicles equipped with multi-vision systems in the automatic valet parking task as an example.

The remainder of this work is organized as follows: In Section 2, some related works are described. In Section 3, we describe our system architecture, followed by semantic information extraction in Section 4. After that, we present the semantic-aware global orientation estimation method in Section 5, with the experiments presented in Section 6 and the conclusion in Section 7.

## 2. Related Works

### 2.1. Multi-Camera SLAM System

In general, increasing the number of cameras in a mobile robot system can effectively improve its perception range, thereby increasing the potential improvement of its perception ability [25,26]. Therefore, researchers are paying more and more attention to how to model multi-vision systems and use the characteristics of multi-vision systems to improve the accuracy and robustness of the system, and the relevant research results have been widely used in mobile robot SLAM tasks [27,28,29].

Although direct processing of multi-camera images maximizes the use of the information in the original image, it also means that sufficient computing resources are required to enable fast processing, so it may not be suitable for tasks that require high real-time performance. As an alternative scheme, some recent research has also been performed to use the image after surround view synthesizing as the input, which can greatly improve the processing efficiency of the system while achieving satisfactory accuracy.

### 2.2. Feature Extraction Techniques for SLAM

The environmental feature information utilized in traditional visual SLAM frameworks typically includes sparse corner features (e.g., SIFT, ORB) in indirect methods and pixel luminance information in direct methods [30,31,32]. On top of this, there are many ways to further improve SLAM by detecting features such as geometric elements, such as line segments and planes, in the scene reliability of the system [12,33,34].

With the rapid development of deep learning in recent years, more and more methods based on deep neural networks have been integrated into visual SLAM systems to improve the system’s perception and utilization of environmental information. As the main means of semantic perception, semantic segmentation and object detection networks are widely used in the acquisition of pixel-level and object-level semantic information, respectively, and bring additional semantic and geometric constraints to the SLAM system, especially in scenarios with many dynamic objects, which significantly improves the stability and accuracy of the SLAM system. Semantic SLAM has been widely studied and implemented in various kinds of robots by the substantial progress of deep learning techniques, especially alongside high-performance computing machines, such as graphics processing units (GPUs). For example, the image-segmentation-based neural network models include the FCN [35], SegNet [36], PSPNet [37], ICNet [38], DeepLab [39], MobileNet [40], and their extensions; the image-based object detection network models include RCNN [41], YOLO [42], SSD [43], and their extensions; point cloud segmentation network models include PointNet [44] and its extensions; point cloud-based object detection network modes include VoxelNet [45], PointPillars [46], and their extensions. These semantic segmentation and object detection networks have been applied to extract object-level and point-level semantics, which bring prior structures into the SLAM system, and more accurate and robust performances are obtained. However, these deep-network-based methods need huge amounts of labeled data, which are usually not affordable for practical applications. Therefore, we propose a hybrid and multi-level information fusion scheme to deal with this problem.

### 2.3. Semantic Information with Synthesized Surround View Image

The homogeneous transform or IPM relied on by surround view synthesizing techniques is a very classic image processing method [47,48]. With the rapid development of vision systems in recent years, this technology has also been widely used, such as the reverse assistance system in cars, which usually uses this technology to enable the driver to easily observe the situation of surrounding objects and the distance from the robot. Similarly, when mobile robots complete tasks such as mapping, positioning, and navigation, researchers also focus on how to extract effective semantic information from the synthesized surround view image, such as various pavement markings, obstacles, and passable areas, and describe them in different forms of representation such as point clouds and occupied grids to help complete related tasks [49,50].

However, because models based on deep neural networks often require a large amount of manually labeled data for training, they are difficult to quickly scale and apply to new scenarios. Therefore, this work considered only the rough passable space segmentation results to assist in extracting the semantic information in the surround view synthesized image and obtain rich and effective semantic information such as passable area boundaries, pavement sparse feature points, and pavement marking edges, which not only improves the effective information quality of the input SLAM system, but also greatly reduces the requirements for annotated data, so it can be applied to mapping positioning and navigation tasks in new scenes more quickly than the previous methods.

### 2.4. Semantic-Feature-Based Global Localization

Due to the high ability of convolutional neural networks to discover complex patterns, NetVLAD uses the network to generate global descriptors directly end-to-end [51]. LoST uses the network to learn local key points, global descriptors, and semantic information to complete VPR tasks with extreme changes in view and appearance [52]. In addition, some methods even use the network directly to give the results of pose estimation end-to-end [53,54,55]. Although their results show extremely high accuracy and robustness to noise in the dataset, they are invariably dependent and data-dependent, so the generalization performance of these methods is not satisfactory.

As a high-level feature, semantic information is compact and has good stability, so it is suitable as a reference for visual localization. Some research work used specific landmarks as masks to avoid extracting feature information in some dynamic regions [56]. VLASE uses the semantic edges extracted by the network to characterize the image and, then, achieve localization. Some methods use columnar landmarks as a special location reference to improve the positioning accuracy of robots [57]. The aforementioned methods only consider the information of the episemantic class. However, the spatial relationship between semantics also implies information about the place. Therefore, the method proposed in this work uses both episemantic and spatial distribution information to pursue a complete description of the scene. Some studies use graph models to encode scenes and their topologies, but building graphs from images is a difficult problem, especially in sparse landmark scenes [58,59]. In this work, semantic information is explicitly encoded as semantic vectors, and spatial geometric relationships are represented in the form of histograms. The descriptor constructed by this encoding method has a compact structure and good interpretability, which is commonly used in various scenarios.

## 3. Structure-Aware Global Orientation Estimation System

**Figure 1:**
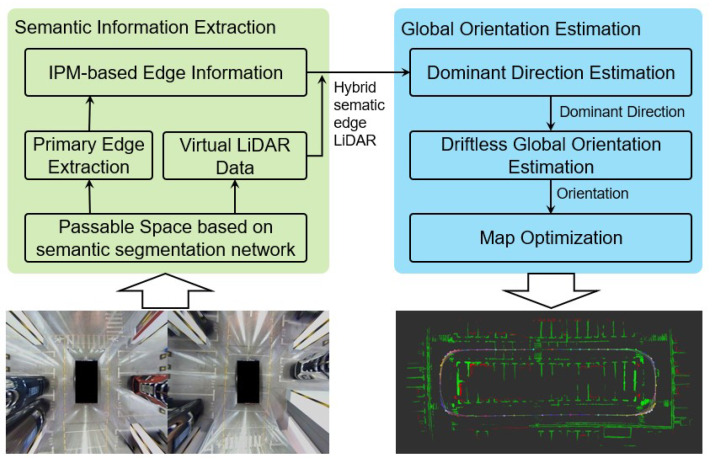
Semantic-structure-aware global orientation estimation framework.

In this work, we built a SLAM framework based on semantic-aware structural information for estimating the robot’s global orientation state, as shown in Figure 1. The framework takes a synthesized surround view image as the input and outputs a drift-free trajectory and a map consisting of marker lines. As we can see, the proposed SLAM system consists mainly of two procedures, structured semantic information extraction and orientation estimation. During the structured semantic information extraction stage, the passable space in the image is firstly extracted with the help of the semantic segmentation network model as a mask for feature extraction; thus, we can improve the computational efficiency of the system while retaining most of the effective information. Since the boundary of the passable area contains geometric information about the environment, it could also be converted into a point cloud representation by LiDAR, which effectively complements the visual feature point information. In addition, since the passable space contains rich pavement marking edges, by further using the distortion characteristics of the reverse-perspective projection process, the distortion edges could be effectively eliminated, and the hybrid edge information could be used for mapping and localization tasks. During the orientation estimation stage, based on the dominant orientation information, the global orientation of each frame can be preliminarily estimated without drift. In order to further improve the anti-interference performance, we built a local map and designed it to optimize the state factor in the local map using the global orientation error constraint. Finally, the estimation of the global orientation is obtained, and a line map that reflects the structure of the real scene can be reconstructed.

## 4. Semantic Information Extraction

### 4.1. Virtual LiDAR Data Generation

Conventionally, LiDAR can accurately measure the distance information of objects in the environment relative to the robot itself, and the virtual radar proposed in this work obtains similar distance measurement information through the secondary processing of semantic segmentation results, simulating the detection results of LiDAR sensors. Since most of the area in the original image is the ground and objects on the ground, we considered using IPM to convert the original image to a top view and synthesized it to obtain a surveillance-synthesized image. The process of IPM transformation can be described as

(1) $$\left[\begin{array}{c} x_{veh}\\ y_{veh}\\ 1 \end{array}\right]=\lambda {\left[\begin{array}{cc} R_{c} & t_{c} \end{array}\right]}_{\mathrm{col}:1,2,4}^{-1}\pi_{c}^{-1}\left(\left[\begin{array}{c} u_{fish}\\ v_{fish}\\ 1 \end{array}\right]\right),$$

where ${\left[u_{fish},v_{fish}\right]}^{T}$ denotes the pixel coordinates of the fish-eye camera image, $\pi_{c}\left(\cdot \right)$ denotes the projection model with its inverse projection model as $\pi_{c}^{-1}\left(\cdot \right)$, $\left[\begin{array}{cc} R_{c} & t_{c} \end{array}\right]$ denotes the external parameters of the camera, i.e., the homogeneous transformation matrix from the robot coordinate system to the camera coordinate system, and ${\left[\right]}_{\mathrm{col}\;:k}$ denotes the *k*-th column of the matrix. λ denotes the scaling factor, which can be obtained by calibrating the correspondence between the transformed image and the actual scene size, and ${\left[\begin{array}{cc} x_{\mathrm{veh}\;} & y_{veh} \end{array}\right]}^{T}$ denotes the position of the point in the final robot coordinate system.

After IPM transformation, a virtual camera directly above the robot and shot vertically downward can be further constructed, which can map the points under the obtained robot coordinate system to obtain the final synthesized surround view image as

(2) $$\left[\begin{array}{c} u_{ipm}\\ v_{ipm}\\ 1 \end{array}\right]=K_{ipm}\left[\begin{array}{c} x_{veh}\\ y_{veh}\\ 1 \end{array}\right],$$

where ${\left[\begin{array}{cc} u_{ipm} & v_{ipm} \end{array}\right]}^{T}$ denotes the pixel coordinate of the synthesized surround view image and $K_{ipm}$ denotes the internal parameter matrix for the virtual camera.

**Figure 2:**
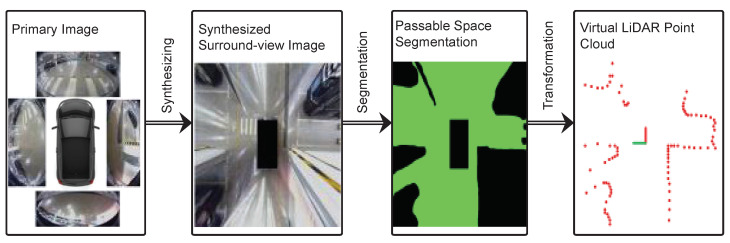
The process of virtual LiDAR data generation.

To obtain the segmentation results of free spaces, this paper trained a semantic segmentation network model to distinguish between passable space and non-passable space. Then, through the morphological processing at the image level, the segmentation results can be further modified to obtain a more ideal segmentation effect, and the boundaries of the passable area can be further extracted. Since the scale transformation coefficient between the pixel distance of the image and the actual distance can also be obtained at the same time during the calibration process of surround view synthesizing, the pixel distance between the point on the passable area boundary in the image and the pixel distance of the image center can be directly converted into the actual distance under the robot coordinate system or virtual radar sensor coordinate system. Then, according to the scanning method using LiDAR, all boundary points are sampled at fixed angular intervals, and the boundary points in the same angle window can be represented by the closest point so that the final virtual radar measurement data can be obtained. The process of virtual LiDAR data generation is shown in Figure 2.

### 4.2. IPM-Based Hybrid Semantic Edge Extraction

**Figure 3:**
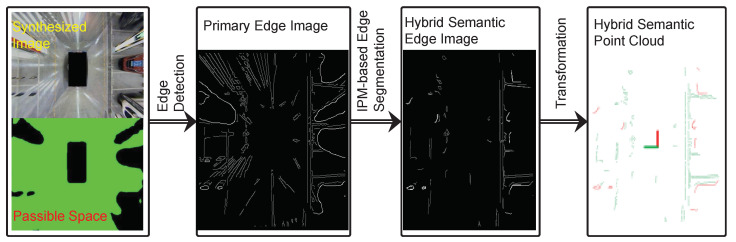
The procedure of hybrid semantic edges extraction.

The original synthesized surround view image often contains a large number of ground markings, which can be fed into SLAM systems as high-quality road sign information. However, the information of the synthesized surround view image is also disturbed by a large number of ground spots. At the same time, the reverse-perspective projection transformation during the synthesizing process will distort objects with a certain height on the ground. Therefore, an edge segmentation module needs to be designed to reject the above interference edges, so that the high-quality effective edges on the ground can be retained. The process of hybrid semantic edge extraction is shown in Figure 3.

Primary edge extraction: The edges on the input surround view synthesized image can be extracted by traditional edge detection methods, such as the Canny edge detector and LSD detector. On the one hand, the edge of the ground mark and the projected edge of the object can be well extracted; on the other hand, traditional edge detectors will also detect those invalid edges, such as the edges of surrounding robots, pillars, and light spots, so the original edges need to be processed to some extent. Due to the segmentation of the passable space, edges that are located on objects above the ground level can be easily removed. However, there is still a considerable part of the spot edge that cannot be removed in this way, and the part of the passable space boundary that is affected by distortion requires additional processing; otherwise, it will not be able to be entered into the subsequent SLAM system and achieve the ideal positioning and mapping results.

IPM-based edge segmentation: Considering that the distortion edges are mainly centered on the photocenter of each camera and distributed in the direction of the rays, an intuitive idea is to build a polar coordinate system with its photocenter as the origin for each camera and count the number of edge points in each direction. However, the segmentation method based on ray accumulation has problems such as erroneous removal of dense small edges, such as the edge of zebra crossings, threshold coupling of angle parameters and the number of edge points in the fan, and inaccurate results. In order to consider the geometric distribution of edges, further attempts can be made to detect line segments in the edge image. Specifically, traditional linear detectors, such as those based on the Hough transform, can extract segments of a length from the original edge image, then calculate the distance between the line in which each segment is located and the camera center of its field of view. Finally, those segments that are small enough away are marked as distorted edge areas. However, the segmentation method based on segment detection has problems such as the erroneous exclusion of some unconnected edge points, and the distance between the line segment and the optical center will also affect the selection of the final distance threshold.

To further consider the fine structure of the edge, different consecutive edges can first be distinguished before edge segmentation. Then, for each edge, the Douglas–Peucker algorithm is used to approximate the edges. The line segments obtained by approximating the contour of each edge are connected to the center of the camera photocenter in the field of view, and then, the angle between the line and the line segment is calculated to remove those parts whose angles are less than a certain threshold. The main advantage of the segmentation method based on polyline approximation is that it simplifies the operation of segment detection and limits segment approximation to the interior of each edge. At the same time, for edges at different distances, the way of evaluating the angle is more stable and consistent than the previous way of evaluating the distance, especially for those edges that are very far from the robot. The algorithm of the polyline-approximation-based edge segmentation method is shown in Algorithm 1.
**Algorithm 1** Polyline-approximation-based edge segmentation algorithm.1:**Input:** Synthesized surround view image within the passable area $I_{\mathrm{edge}}$.2:**Output:** Valid edge image after segmentation $I_{\mathrm{seg}}$.3:**Initializing:** The maximum allowable error of the polyline approximation $D_{max}$, the maximum allowable angle threshold for the line and polyline between the midpoint of the effective polyline and the camera optical center $\theta_{max}$, reject range *r*.4:Extracting the contours of all edges in $I_{\mathrm{edge}}$ to form the edge profile set C;5:**for** c∈C **do**6:    Computing the polyline set L by taking advantage of Douglas–Peucker operators and $D_{max}$ to approximate the contours with polylines;7:    **for** l∈L **do**8:        Computing the camera center note position *C* of the field of view based on the endpoint of the polyline *l*;9:        Computing the connecting line $l_{C}$ between the middle point of *l* and the camera optical center *C*;10:        Computing the angle θ between *l* and $l_{C}$;11:        **if** $\theta <\theta_{\max}$ **then**12:           Removing all the edge points covered by *l* along with reject range *r* from $I_{\mathrm{seg}}$;13:        **end if**14:    **end for**15:**end for**

## 5. Semantic-Aware Global Orientation Estimation

### 5.1. Local Dominant Direction Estimation

**Figure 4:**
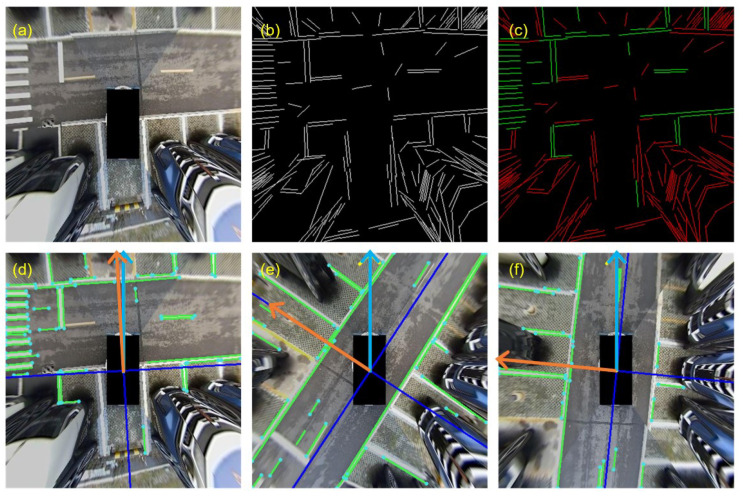
The three subfigures in the upper row illustrate the procedure of structure information extraction: (**a**) denotes the raw input synthesized image, (**b**) the extracted raw lines, and (**c**) the segmentation results, in which green lines denote the preserved marking lines and red lines denote the noisy lines. (**d**–**f**) denote the relationship between the local dominant direction and global orientation, in which the orange arrow denotes the local dominant direction and the blue arrow denotes the global dominant direction for the current frame, respectively, while the green lines denote the marking lines.

In a real navigation environment, there is a large number of road markings on the ground, which is a good reference landmark for SLAM systems. However, there are also various disturbing noises on the ground, such as glare or water stains. Therefore, it is necessary to distinguish the marker lines from the noise lines. By observation, it can be seen that most of the marker lines are parallel or perpendicular to each other, while the noise lines are disordered. Inspired by this phenomenon, this section designs a segmentation method for marker lines based on the structural rules of the Manhattan world, which can be used to obtain from the image a collection of line segments that conform to the structural assumptions of Manhattan and the dominant orientation of these lines, as shown in Figure 4. The left (a) is the raw input image. The middle (b) is the extracted raw lines, and the right (c) is the line segmentation result. Green lines are the preserved marking lines, and red lines are the noisy lines. The bottom row shows three consecutive images. Green lines are marking lines; the orange arrow is the local dominant direction of the current frame, and the blue arrow is the global dominant direction.

In the initial frame, the line with the highest number of perpendicular and parallel to the other lines is considered to be the initial dominant direction $x_{d}$, and the vertical or parallel between two lines can be evaluated by computing,

(3) $$\theta -\arccos\left(\frac{x_{i}\cdot x_{j}}{\left|x_{i}\right|\left|x_{j}\right|}\right)<\delta ,$$

where δ denotes the tolerance and θ can be set as 0 for the parallel lines’ evaluation and $\frac{\pi }{2}$ for the perpendicular lines’ evaluation. Thus, we can build two sets for these two types of lines as $L_{//},L_{⊥}$ for parallel lines and perpendicular lines, respectively. Then, we optimize the dominant direction:

(4) $$\min_{a,b}\sum_{l_{i}\in L_{//}}\left|x_{d}^{\prime }\cdot l_{i}\right|+\sum_{l_{j}\in L_{⊥}}\left|x_{d}\cdot l_{j}\right|.$$

The first term denotes that the parallel line intersects with the dominant direction at point $x_{d}^{\prime }=\left[b,-a,0\right]$, and the second term means the perpendicular line intersects with the dominant direction at point $x_{d}=\left[a,b,0\right]$.

Due to the robot’s dynamic constraints, the maximum directional change in adjacent frames is assumed to be η. After successful initialization, in order to reduce the amount of calculation, subsequent frames will only look for a new dominant direction and the corresponding set of structure lines from candidate line features in the interval with the dominant direction of the previous frame less than η.

### 5.2. Global Orientation Optimization

When a new image appears, first extract the marker line collection and determine the local dominant direction according to the method described in the subsection before. As shown in Figure 4, the orientation of the current frame equals that angle between the global orientation and the dominant direction of the current frame; thus, it can be computed as

(5) $$\theta_{z}=\arccos\left(\frac{I_{g}\cdot I_{c}}{\left|I_{g}\right|\left|I_{c}\right|}\right),$$

where $I_{g}$ denotes the global orientation and $I_{c}$ denotes the dominant direction of the current frame.

A collection of marker lines may contains some noise lines. However, these noise lines also satisfy the geometric rules of the Manhattan world assumption, so they have little effect on the orientation estimates. This way of estimating orientation is independent of the orientation estimation of adjacent frames, so there is no cumulative error. When the orientation is known, only the translation term remains to be estimated. In this way, the originally nonlinear pose estimation problem is transformed into a linear least-squares problem.

Due to the existence of occlusion, visual blurring, and other factors, the consistency of line segment detection is poor, and a line segment is often split into two independent line segments, or the length of the line segment will change. This results in the line feature not binding enough on the amount of translation in the extension direction and may even introduce incorrect constraints. Although it is also possible to extract the features of points separately to constrain the amount of translation, this will cost additional computational resources. As shown in Figure 4, the endpoints of many segments are also corner points. Therefore, in order to solve the above problem, this paper takes the endpoints of the line segment as point features and uses bidirectional optical flow to trace these endpoints to establish data associations between endpoints, rather than between line features. More specifically, mapping the feature points from the reference frame to the current frame, denoted as $P_{r}$, re-mapping the feature points from the current frame to the reference frame, denoted as $P_{c}$, only if enough point pairs are obtained, we can compute the translation term as

(6) $${}^{W}t_{B_{i}}={}^{W}R_{B_{j}}\cdot {\bar{p}}_{B_{j}}+{}^{W}t_{B_{j}}-{}^{W}R_{B_{i}}\cdot {\bar{p}}_{B_{i}},$$

where $\bar{p}$ denotes the mean position of the point sets. Then, estimate the translation of the current frame via the reprojection errors.

If the current frame is more than 10 frames away from the previous keyframe and the required number of features is met, the current frame is selected as the keyframe and added to the local map. Then, optimize the local map using an objective function that minimizes the global orientation residuals and reprojection errors as

(7) $$\min_{{}^{W}T_{B_{i}}}w\sum_{i}\left(1-\frac{I_{i}\cdot \left({}^{B_{i}}R_{W}I_{g}\right)}{\left|I_{i}\right|{\left|\right|}^{B_{i}}R_{W}I_{g}|}\right)+\sum_{i}{∥p_{i}-{}^{W}T_{B_{i}}\pi_{s}^{-1}\left(u_{i}\right)∥}_{2},$$

where the former term means the global orientation residual errors since the angle between $I_{i}$ and ${}^{B_{i}}R_{W}I_{g}$ should be zero. ${}^{B_{i}}R_{W}$ denotes the transformation of $x_{g}$ from the world coordinates to the robot frame *B*. The latter term means the reprojection errors. ${}^{W}T_{B_{i}}=\left[{}^{W}R_{B_{i}},{}^{W}t_{B_{i}}\right]\in \mathrm{SE}\left(2\right)$, and $\pi_{s}^{-1}$ transform the pixel $u_{i}$ to the world coordinates *W*. Here, the end-to-line reprojection error commonly used by other methods is not used because the point-to-point reprojection error is more constrained and accurate in the amount of translation.

After local map optimization, the global orientation can be found, which is not disturbed by the accumulated errors. At the same time, the position of the line in the world coordinate system is also determined. Because line features are primarily marked lines on the pavement, maps reconstructed from line features reflect the structure of the road and the markings on the roads.

## 6. Experimental Results

### 6.1. Experiments’ Configurations

**Figure 5:**
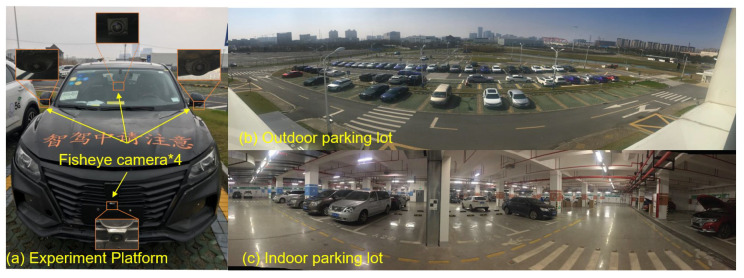
(**a**) The experimental platform with four fisheye cameras. (**b**) The outdoor and (**c**) indoor parking scenes.

In this experiment, an automotive platform equipped with four fisheye cameras was mounted for parking assistance purposes, as shown in Figure 5. The experimental data were recorded by a car and wheel speedometer equipped with four fisheye cameras. All cameras have a 190-degree viewing angle, take images at a frequency of 25 Hz, and have an image resolution of 1920*1208. Each camera was connected to the in-vehicle computing platform NVIDIA Drive PX2 via a Gigabit multimedia serial interface. Timestamp synchronization is guaranteed by the hardware. The composite ring view has a resolution of 384*384 and covers a range of 15.3 m by 15.3 m around the vehicle circumference. The measured values of the wheel speedometer and IMU are obtained via the CAN bus. The experimental computing platform has a configuration of 3.3 GHz Intel i5-4590 CPU and 16 GB memory. In the indoor scenes with weak GPS signals, the true value of panning is provided by the fusion of wheel odometry and high-precision IMU measurements. In the outdoor scenes, the true value of planning is provided by the high-precision differential GPS.

### 6.2. Evaluation of Structural Information

**Figure 6:**
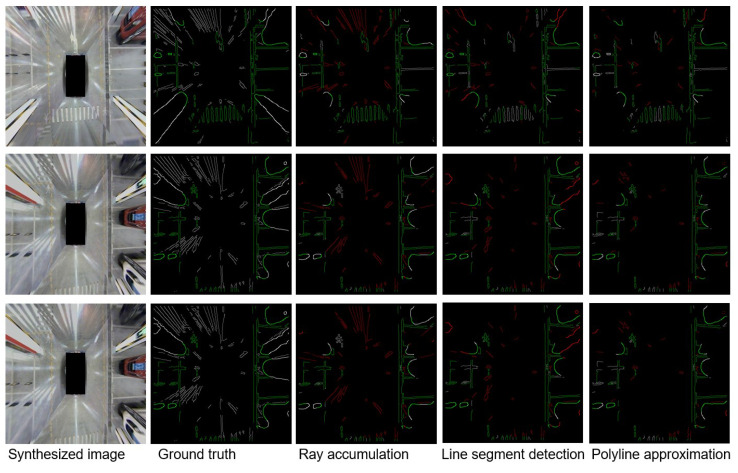
The comparison of different edge segmentation methods. Each row represents the results for different structure extraction methods for the same synthesized image, which is illustrated in the first column, and each column represents the results for different synthesized images with the same structure information extraction method, which is described at the bottom of each column. The first column denotes the three input images, the second column the ground truth of edge information, the third column the edge information extracted by the ray accumulation method, the fourth column the edge information extracted by the line segment detection method, and the fifth column the edge information extracted by polyline approximation method. The manually labeled ground truth edges are drawn in green. For each column of segmented edges, the green lines denote that the edges are correctly preserved, while the red lines denote that the edges are not correctly preserved, and the white lines denote those missed for the specific method. Moreover, The color intensity means whether the edges are inside the free space (brighter) or on the contour of the free space (darker).

Figure 6 shows some sample results of different edge segmentation methods at the same level of recall. For the original edge picture corresponding to each test sample, the truth value of the edge segmentation result can be given by manual annotation, and then, the difference between the segmentation result of each method and the true value can be compared, including the part that was correctly retained, the part that was mistakenly rejected, and the part that was incorrectly retained. As we can see, the splitting method based on ray accumulation retains too many wrong spot edges, usually because the same long edge is incorrectly divided into different sector areas, and the length of each segment does not reach the set threshold, so it cannot be correctly rejected. The segmentation method based on line segment detection can detect most of the spot edges, but for the less straight edges in the distance, especially the boundaries of the passable area in the distance, the set distance threshold cannot be reached, so it will be incorrectly retained. The segmentation method based on polyline approximation can successfully remove most of the distorted edges, while only a small number of effective edges are erroneously rejected because they are exactly in the direction of the camera’s field of view rays.

More specifically, as shown in Table 1, when the recall of all methods is controlled at about 0.73, the segmentation method based on polyline approximation can achieve the best segmentation effect, which is 24.3 percent higher than the segmentation method based on ray accumulation and 11.9 percent higher than the segmentation method based on line segment detection.

### 6.3. Global Orientation Optimization

**Figure 7:**
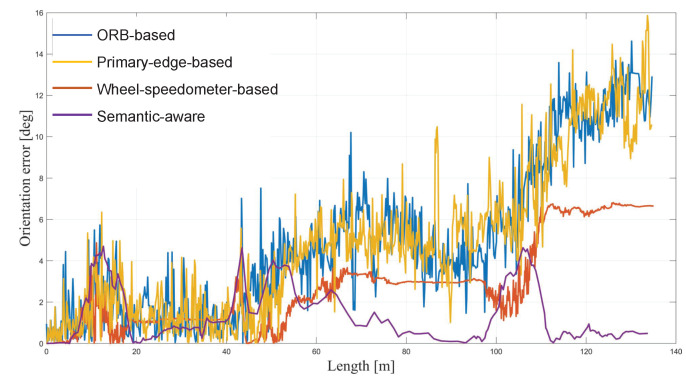
The comparisons of the orientation estimation errors of different methods.

We compared our semantic-structure-based method (semantic-aware) with three other methods, ORB feature-point-based method (ORB-based), primary-edge-based method (primary-edge-based), and wheel-speedometer-based method (wheel-speedometer-based). In Figure 7, we show the comparisons of the orientation estimation errors for the different methods. As the trajectory becomes longer, the orientation angle error of the three comparison methods gradually increases. The main reason is that errors accumulate over time. However, our method achieves a global estimation of the change in direction and avoids the occurrence of accumulated errors. Therefore, the orientation error of this algorithm is independent of the length of the trajectory. When the vehicle turns, the orientation error of all methods increases significantly. This is mainly caused by blurry images caused by fast rotation. Table 2 shows a comparison of the mean orientation estimation errors of the different methods. The average orientation estimation error of the methods presented in this work is stable between 1 and 2 degrees, well below other methods, both indoors and outdoors. This also verifies that, among the different types of feature information, the structure line information is the most robust to the interference factors, and the orientation results estimated based on it are the most accurate.

**Figure 8:**
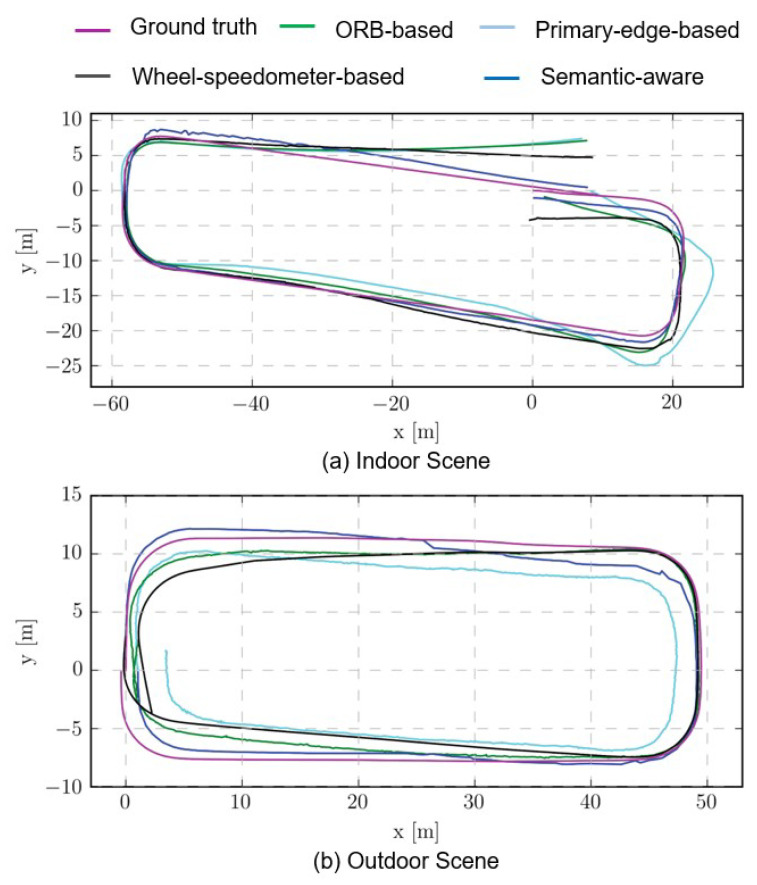
The comparisons of the trajectory estimation for different methods. (**a**) Indoor Scene, (**b**) Outdoor Scene.

The comparisons of the trajectories for the different methods are shown in Figure 8. Since the proposed method has the most-accurate orientation estimation, the trajectory drift value of the proposed method is much smaller than that of other methods. The trajectory estimated using other information has a significant drift. At the same time, it can be seen from the trajectory comparison chart that, although the trajectory using structural information is more consistent with the true value in the upward direction, there are also jagged oscillations and even mutations in some local areas. This is actually due to environmental factors: when there are few marked lines on the road surface in the environment and when the line features are not accurate enough due to blurry images, the estimated dominant direction will oscillate, rather than be smooth enough. Therefore, although the positioning strategy based on structural information proposed in this paper performs well in orientation estimation, the overall trajectory still has errors compared with the truth trajectory.

## 7. Conclusions

This paper used high-level semantic information and spatial distribution information to assist the visual location recognition task and used the semantic structure information to realize the global orientation estimation method without drift interference. In this paper, a visual semantic perception system based on the synthesized surround view image was proposed for the multi-eye surround vision system, which was used to obtain the visual semantic information required for SLAM tasks. Different from the traditional method of obtaining feature information from the original multi-eye image, the original multi-eye image was transformed and synthesized by reverse-perspective projection to obtain a synthesized surround view image that could describe the scene in an all-round way, to improve the computational efficiency of the system while retaining most of the effective information. To retain the effective information in the synthesized surround view image and remove the features that had been distorted during the synthesizing process or located on the dynamic object, this paper extracted the passable space in the image with the help of the semantic segmentation network model as a mask for feature extraction. Since the boundary of the passable area contained geometric information about the environment, it could also be converted into a point cloud representation by LiDAR, which effectively complements the visual feature point information. In addition, since the passable space contained rich pavement marking edges, by further using the distortion characteristics of the reverse perspective projection process, the distortion edges could be effectively eliminated, and the hybrid edge information could be used for mapping and localization tasks. The experiments based on the indoor and outdoor automated valet parking verify that the proposed scheme can achieve more precise edge segmentation results, much smaller orientation estimation error, and better trajectory estimation. In the future, we hope to investigate the generalization of our algorithm in more diverse scenarios, such as light changes and more dynamic tasks.

## Figures and Tables

**Table 1:** Comparisons of the precision and recall rates among different edge segmentation methods.

Methods	Precision	Recall
Ray-accumulation-based segmentation	0.621	0.731
Line-segment-detection-based segmentation	0.745	0.729
Polyline-approximation-based segmentation	0.864	0.730

**Table 2:** Comparison of the orientation estimation errors of different methods.

Methods	ORB-Based (°)	Primary-Edge-Based (°)	Wheel-Speedometer-Based (°)	Semantic-Aware (°)
Outdoor-navigation	6.426	5.885	3.815	1.491
Indoor-navigation	4.839	4.342	3.095	1.897

## Data Availability

Not applicable.
